# The Toxic Origin of Brain Disease

**Published:** 1901-07-06

**Authors:** 


### THE TOXIC ORIGIN OF BRAIN DISEASE.

It certainly is curious to notice how rapidly and in how many directions the view of the toxic origin of disease, and especially of processes of degeneration, has of late taken hold of the medical mind. Naturally as a corollary one has to admit that certain tissues have a special aptitude for absorbing these poisons, one an affinity for one kind, another for another, and so forth. But admitting the possibility of this selective action—and who, in view of what happens in plumbism, can doubt it?—we have next to discover on the one hand the nature of the toxins and on the other the nature of the predisposition which enables them to produce effects in some cases which they do not produce in others. That dyspepsias and derangements of the intestinal functions can, and very often do, interfere with the proper action of the mind is a thing within the ken of everyone who has to think; and that under the sway of indigestions of various kinds—which are but the brewings of toxins—temper may give way is common knowledge to all whether they think or not; and it is no very great extension of the hypothesis which would explain these things on a humoral basis to recognise that the continued presence of certain toxins—sometimes, as with lead and arsenic, definite metallic poisons, sometimes, as in diphtheria, the product of microbic growth, may lead not merely to temporary disturbance of function, but to actual degeneration of the particular tracts of nerve tissues between which and these several poisons there exists a selective affinity. Dr. Lewis C. Bruce, writing in the *British Medical Journal*, June 29, on the cause of general paralysis of the insane, comes to the conclusion that this disease is directly due to poisoning by the toxins of bacteria, whose point of attack is through the gastric and intestinal mucous membrane, and that although it is probably a case of mixed poisoning the bacillus coli is apparently one of the noxious organisms. Dr. Ford Robertson, working at the same subject, and writing in the same journal, also holds that general paralysis is dependent upon the occurrence of a chronic toxæmia of gastro-intestinal origin; but he holds that these do not act directly on the nervous tissues but on the blood vessels by which these tissues are fed, producing in them proliferative and degenerative changes. This toxæmia, he says, is probably in some measure established for months, or even years, before the first symptoms of nervous disease manifest themselves. "In some individuals the cerebral vessels give way earliest, and general paralysis is the result; in others the vessels of the cord are the more vulnerable; those that receive the largest quantity of blood—and therefore of toxin—suffer first and most severely, and the clinical and pathological picture of tabes dorsalis develops." Dr. Ford Robertson enters into an interesting discussion as to the manner in which these toxins obtain access to the economy, and as to the reasons why they show themselves so especially prone to attack the subjects of old and worn out syphilis, but into these questions we have not space to enter at the present moment. What we wish to point out is the growing tendency to attribute conditions which come before us as organic diseases to the prolonged operation of toxins upon tissues for which they have a selective affinity. In the same journal Professor Haliburton and Dr. John McKendrick describe how in a case of gastric tetany they were able to discover a special toxin, and they express the opinion that a poisonous substance is formed in the stomach, which, when absorbed into the blood stream in sufficient amount, gives rise to the tetanoid contractures and other concomitant symptoms of the disease. Again in the same journal we have a paper by Dr. Hamilton Wright on the changes in the neuronal centres in beri-beric neuritis, which shows how very similar they are to those which occur in such a toxic form of neuritis as that which arises from alcohol. Thus we have a series of papers, each of which has been written to elucidate a given pathological condition, but the summation of which puts in striking prominence the influence of toxins in the production of disease, and, we may add, the readiness of investigators at the present day to seize upon them as the *deus ex machinâ* by which pathological processes are to be explained. The influence of this idea upon treatment is a somewhat different affair. We have to remember that by the time the patients show signs of mental disease it may be, and according to the hypothesis of Dr. Robertson it must be, that organic changes have long ago taken place. Thus the psychologist seeks for other remedies in other fields than in mere intestinal antisepsis. But if it be true that the intestinal canal with its varied microbial flora is the real starting-point of such serious organic diseases as general paralysis and tabes dorsalis, there must be a time, if we did but know it, when the old-fashioned purging and starvation might possibly have exorcised these evil microbes. It has long been a formula with some schoolmasters that when a boy turns non-receptive, the best plan is to give him a dose of castor oil. Even Mr. Squeers found that a mixture of brimstone and treacle was not without effect in restoring harmony when his scholars proved rebellious, and if the auto-intoxication theory which is now coming so much into prominence is to be accepted perhaps they were not altogether wrong. Whether the good old Friday fast is not an even better means of maintaining health of body and equability of mind is another question. At any rate the more we see of the dangers which may arise from our interiors being turned by excess of food into fermenting bacteria beds, the more convinced we become of the advantages of combining high thinking with plain living. For years we have been told that the increase of general paralysis has been due to the over-strain of the nervous system to which civilised people are exposed. Perhaps it may turn out to be due to their overfeeding and to their ill-regulated interiors. At the least this hypothesis is a more hopeful one than that which suggests that if we will be civilised we must put up with a growing percentage of broken down nervous systems.

